# Machine learning models in predictive factors for megaloblastic character of macrocytic anemia

**DOI:** 10.1016/j.lrr.2026.100568

**Published:** 2026-02-17

**Authors:** Melek Kechida, Mohamed Kenani, Nader Slama, Manel Mili, Aminetou Salem, Wassim Krit, Ines Khochtali, Sarra Boukhris, Asma Ben Abdallah, Mohamed Hedi Bedoui

**Affiliations:** a Department of Internal Medicine and Endocrinology, Fattouma Bourguiba University Hospital, Faculty of Medicine of Monastir, University of Monastir, Monastir, Tunisia; b Department of Hematology, Fattouma Bourguiba University Hospital, University of Monastir, Monastir, Tunisia; c Department of Informatics, Faculty of Science of Monastir, University of Monastir, Monastir, Tunisia; d Department of Informatics, Higher Institute of Computer Sciences and Mathematics of Monastir, University of Monastir, Monastir, Tunisia; e Technology and Medical Imaging Laboratory, Faculty of Medicine Monastir, University of Monastir, Monastir, Tunisia

**Keywords:** Megaloblastic, Anemia, Machine learning, Prediction, Macrocytic

## Abstract

**Background:**

Differentiating megaloblastic from non-megaloblastic macrocytic anemia usually relies on vitamin B12 and/or B9 assessment and bone marrow examination. In resource-limited regions, these diagnostic tools are not readily available. This study aims to develop a machine learning model to distinguish between these two forms of macrocytic anemia using commonly accessible clinical and laboratory parameters.

**Methods:**

A logistic regression model was developed using a dataset containing clinical features and routine laboratory data. Key predictive variables were identified through the SequentialFeatureSelector. The model was optimized using GridSearchCV and validated through stratified 5-fold cross-validation to ensure robust performance.

**Results:**

ESR, glossitis, and MCHC were the most influential variables in predicting non-megaloblastic anemia, with respective importance percentages of 23.11 %, 22.07 %, and 13.87 %. Other variables such as LDH (13.77 %), aregenerative status (13.42 %), and limb paresthesia (11.78 %) also contributed significantly to the model, while MCV had a comparatively lower importance (1.98 %). The model demonstrated a high discriminatory ability, with an area under the ROC curve (AUC) of 0.92 and an overall accuracy of 94 %. Learning curve analysis confirmed its stability and consistent performance across varying data sizes.

**Conclusions:**

By reducing dependence on specialized diagnostic tests and invasive procedures, this approach is particularly suited for resource-constrained healthcare settings.

## 1 Introduction

Anemia remains a significant global public health concern, despite notable progress in living standards. It is a predictor of mortality among the elderly and significantly affects their overall quality of life [1].

Macrocytic anemia is notably prevalent among the elderly, ranging between 3.3 % in women and 6.3 % in men aged over 65 [2,3]. The primary form of macrocytic anemia is megaloblastic anemia, typically resulting from vitamin B12 or folate deficiencies. Megaloblastic anemia diagnosis is usually based on bone marrow examination or vitamin B12 and folate levels analysis, generally conducted at specialized centers.

Artificial Intelligence (AI), particularly machine learning (ML), is increasingly being integrated into medical practice, offering innovative tools to enhance diagnosis and therapeutic decision-making across various specialties, including hematology [4]. Among these, logistic regression stands out as a robust and interpretable machine learning method for predictive modeling.

In this study, we aim to develop a logistic regression model capable of distinguishing the megaloblastic origin of macrocytic anemia based on clinical signs and basic biological parameters. Our goal is to rely exclusively on readily available data, circumventing the need for specialized tests, such as vitamin B12 and folate level assessments, which may not be accessible in all healthcare settings. By doing so, we also aim to minimize the reliance on invasive diagnostic procedures like bone marrow aspiration.

## 2 Materials and methods

### 2.1 Study design and data description

We conducted a retrospective descriptive study of patients diagnosed with macrocytic anemia at the Internal Medicine and Hematology Departments of Fattouma Bourguiba University Hospital in Monastir, covering the period from January 1st, 2009 to December 31st, 2023. All patients met the WHO criteria for hemoglobin levels [2] and had an average mean corpuscular volume (MCV) greater than 100 fl. The demographic, clinical, and biological characteristics of the patients were recorded. Patients were divided into two groups: those with megaloblastic anemia and those without.

### 2.2 Tools

In this study, data analysis, model development, and graphical visualization were conducted using Jupyter Notebook version 6.5.3. The programming language used was Python (version 3.11.5), along with several essential libraries. For data manipulation and analysis, we utilized SciPy (version 1.11.1) and scikit-learn (version 1.6.0). The logistic regression model was implemented using scikit-learn, while Matplotlib (version 3.7.2) and Seaborn (version 0.12.2) were employed for creating the visualizations. These tools provided a comprehensive environment for performing data analysis, constructing the predictive model, and generating informative graphics.

### 2.3 Preprocessing

We began by analyzing the variables, expressing categorical variables in terms of frequency and percentage, and continuous variables in terms of mean and standard deviation. We then conducted a univariate analysis of all the variables based on the type of megaloblastic anemia. We studied the distribution and variance of continuous variables, as well as the expected frequency of categorical variables, to select the appropriate statistical test.

#### 2.3.1 The significance level was set at 0.05

We analyzed the missing values for the significant variables. The variables with missing values are relevant parameters and easily available, which is why we decided to perform imputation of the missing values using a nearest neighbor strategy with KNN Imputer, with n_neighbors=5, separately for each group of our target variable. We then repeated the univariate analysis for the modified variables to ensure that this process did not distort our data.

Looking ahead, the major challenge was the class imbalance. Indeed, the 'non-megaloblastic' group represents only 14.83 %. We did not want to risk biasing the model by synthetically generating new data. Therefore, we took several precautions at different stages of the process.

### 2.4 Variable selection

The first step of the process was to select the variables to be included in our model. To do this, we used the Sequential Feature Selector method, in combination with logistic regression. Continuous variables were standardized using a Standard Scaler, which is essential for linear models such as logistic regression. This standardization prevents certain variables from dominating due to differences in scale.

Furthermore, to ensure a robust evaluation and account for class imbalance in our target variable, we chose to split the data into training and test sets using train_test_split, specifying stratification on y. This ensures that the class distribution in both the training and test sets remains similar. To reliably assess the model's performance, we adopted stratified 5-fold cross-validation (Stratified K-Fold). This allowed us to perform a progressive feature selection while optimizing the model based on the recall metric, which is particularly useful in cases of class imbalance, where it is crucial not to miss any positive cases.

The feature selection process helped identify the most influential variables for prediction, reducing the risk of overfitting the model with uninformative variables.

### 2.5 Model creation

The selected variables were then used to fit the final model, aiming to maximize its ability to correctly predict cases of non-megaloblastic anemia.

We used a logistic regression model from scikit-learn. First, we integrated preprocessing for continuous and categorical variables into a pipeline via a Column Transformer. Continuous variables were normalized with a Standard Scaler while categorical variables were binary encoded by replacing 'yes' with 1 and 'no' with 0 directly in the dataset. This pipeline was then integrated into a logistic regression model, and we optimized the model's hyperparameters using Grid Search CV. Although, due to the imbalance in our data, we should have specified the 'class_weight' hyperparameter as 'balanced', we preferred to include it along with the other hyperparameters in a param_grid dictionary when using Grid Search CV. We tested the use of class weight (balanced or None), the type of regularization (L2 or None), the C parameter, and the solver. The model was optimized with stratified 5-fold cross-validation. Once the best parameters were identified, we trained the model and made predictions on the test data.

### 2.6 Model evaluation

To evaluate its performance, we used the Receiver Operating Characteristic (ROC) curve and calculated the Area Under the Curve (AUC). We also determined an optimal threshold by maximizing the difference between the true positive rate and the false positive rate, which allowed us to adjust the final predictions. A confusion matrix and a classification report were generated, and a learning curve was produced to visualize the stability of the model with different training sample sizes.

### 2.7 Commitment to open science

To ensure complete transparency and foster collaboration within the medical and scientific community, we have made all our resources publicly available online. This includes the dataset used for model development, the code employed in building the predictive application, and the source code for the application itself. These resources can be accessed via the following link: https://github.com/DrKenani/megaloblastic_anemia.

## 3 Results

### 3.1 Summary of significant variables and management of missing data

**Fig. 1:**
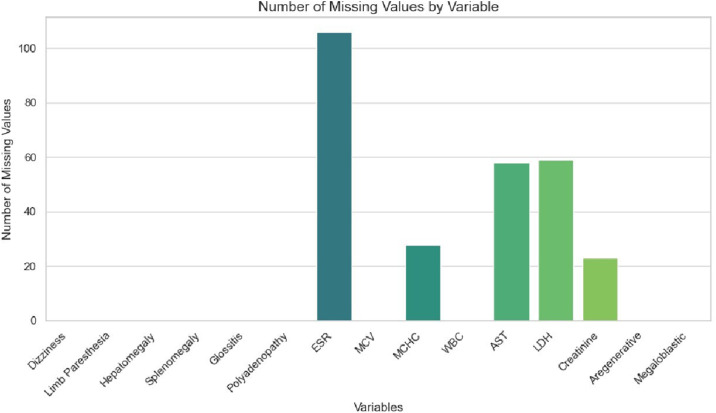
Number of missing values by variable.

AST: Aspartate transaminase; ESR: erythrocyte sedimentation rate; LDH:Lactate dehydrogenase; MCV: mean corpuscular volume; MCHC: mean corpuscular hemoglobin concentration; WBC: white blood cells

Our sample consisted of 263 individuals, with a mean age of 61 years (ranging from 16 to 90 years) and no significant differences based on age and gender. Mean hemoglobin level was 7.38 (±2.35)g/dl. The Table 1 presents the significant variables in univariate analysis based on whether the anemia is megaloblastic or not. The first column describes the entire population, followed by the results of the univariate analysis before imputation of missing values, and then after the imputation of missing values. Only the variables that showed statistical significance were included in this table, with the exception of sex and age, which were displayed to show that there is no significant difference based on these two variables. An additional graph Fig. 1 illustrates the number of missing values for each of these variables.

**Table 1:** Analysis of significant variables according to the anemia group.

		before imputation of missing values				after imputation of missing values		
variable		Descriptif (*n* = 263)	non-megaloblastic anemia (*n* = 39)	megaloblastic anemia (*n* = 224)	p_value	non-megaloblastic anemia (*n* = 39)	megaloblastic anemia (*n* = 224)	p_value
**sexe**	**female**	131 (49.81 %)	19 (48.72 %)	112 (50.00 %)	1.000			
	**male**	132 (50.19 %)	20 (51.28 %)	112 (50.00 %)				
**age**		61.02 ± 15.97	64.95 ± 16.97	60.33 ± 15.73	0.087			
**Dizziness**		115 (43.73 %)	9 (23.08 %)	106 (47.32 %)	0.008			
**Cranial Nerve Involvement**		4 (1.52 %)	3 (7.69 %)	1 (0.45 %)	0.007			
**Limb Paresthesia**		58 (22.05 %)	2 (5.13 %)	56 (25.00 %)	0.011			
**Hepatomegaly**		12 (4.56 %)	5 (12.82 %)	7 (3.12 %)	024			
**Splenomegaly**		17 (6.46 %)	8 (20.51 %)	9 (4.02 %)	<0.001			
**Glossitis**		100 (38.02 %)	4 (10.26 %)	96 (42.86 %)	<0.001			
**Polyadenopathy**		5 (1.90 %)	5 (12.82 %)	0 (0.00 %)	<0.001			
**MCV**		113.69 ± 10.96	106.87 ± 5.15	114.87 ± 11.27	<0.001			
**MCHC**		35.55 ± 5.07	33.42 ± 3.03	35.85 ± 5.23	0.030	33.72 ± 2.67	35.88 ± 5.08	0.033
**WBC**		6421.14 ± 12,704.35	15,323.85 ± 31,216.31	4871.12 ± 2702.20	0.023			
**ESR**		57.41 ± 41.63	92.85 ± 48.30	52.24 ± 38.08	<0.001	92.98 ± 35.04	51.11 ± 31.19	<0.001
**AST**		36.28 ± 35.70	27.63 ± 28.48	38.07 ± 36.84	0.008	28.31 ± 27.14	36.70 ± 32.63	0.005
**LDH**		1628.00 ± 1908.61	816.19 ± 1379.64	1773.46 ± 1956.34	0.001	864.99 ± 1261.57	1753.86 ± 1761.21	<0.001
**Creatinine**		83.02 ± 50.57	109.95 ± 103.77	77.96 ± 29.92	0.006	109.79 ± 102.40	78.10 ± 28.65	0.004
**Aregenerative**		238 (90.49 %)	29 (74.36 %)	209 (93.30 %)	0.001			

**AST**: Aspartate transaminase ; **ESR** : erythrocyte sedimentation rate; **LDH**: lactate dehydrogenase; **MCV**: mean corpuscular volume; **MCHC**: mean corpuscular hemoglobin concentration; **WBC** : white blood cells.

### 3.2 Features selection

Using the SequentialFeatureSelector method combined with logistic regression, we identified the most influential variables for predicting non-megaloblastic anemia. This process, which involved standardizing continuous variables, stratified cross-validation, and optimization based on the recall metric, highlighted the following selected variables : 'ESR', 'Glossitis', 'MCHC', 'LDH', 'Aregenerative', 'Limb Paresthesia', 'MCV'.

### 3.3 Model creation

The logistic regression model, optimized through GridSearchCV, identified the best configuration as *C* = 0.1, class_weight='balanced', penalty='l2′, and solver=’newton-cg’. This choice effectively addressed the imbalance in the target variable by weighting the classes appropriately. The model was trained and validated using stratified 5-fold cross-validation, ensuring robust performance on the test data.

## 4 Final results

The performance of the final logistic regression model is summarized below.

**Fig. 2:**
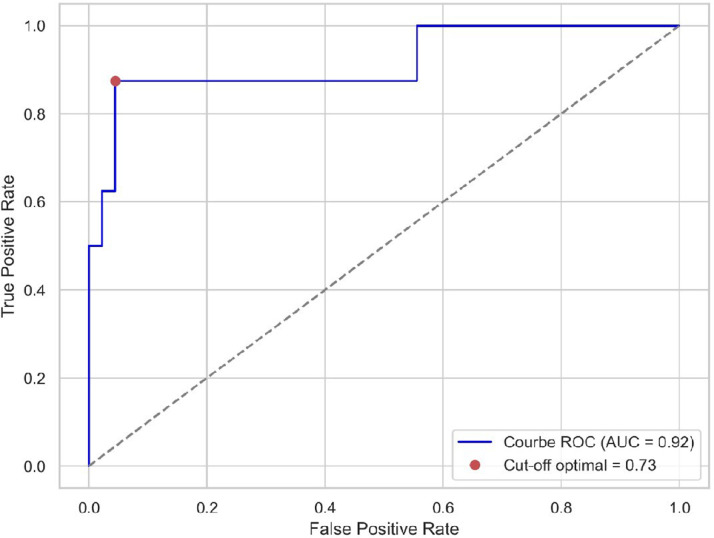
ROC curve.

The ROC curve Fig. 2 demonstrates the model’s ability to distinguish between the two classes, with an Area Under the Curve (AUC) of 0.92. The optimal cutoff point, identified as 0.73, was determined to maximize the balance between sensitivity and specificity.

**Fig. 3:**
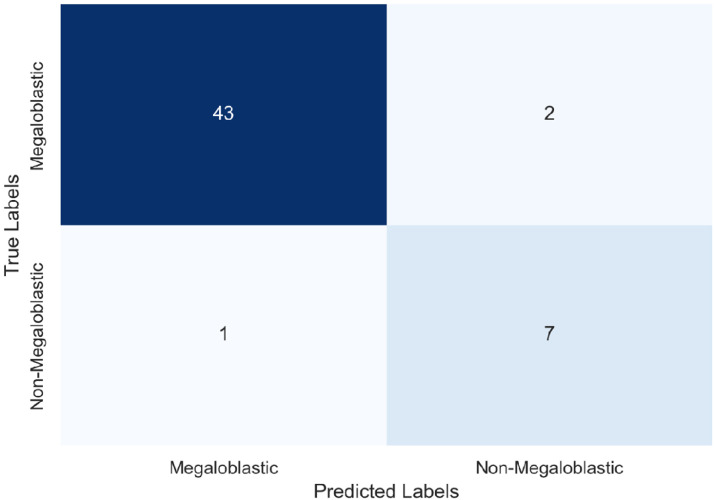
confusion matrix on the data.

The confusion matrix Fig. 3 highlights the classification outcomes for the test data. The model achieved 43 true negatives (correctly identifying megaloblastic cases) and 7 true positives (correctly predicting non-megaloblastic cases), with only one false negative and one false positive.

The model’s overall accuracy was 94 %. Precision, recall, and F1-scores were 0.98, 0.96, and 0.97 for class 0 (megaloblastic) and 0.78, 0.88, and 0.82 for class 1 (non-megaloblastic), respectively, indicating strong performance across metrics Table 2 .

**Table 2:** Classification report.

	precision	recall	f1-score	support
0	0.98	0.96	0.97	45
1	0.78	0.88	0.82	8
accuracy			0.94	53
macro avg	0.88	0.92	0.89	53
weighted avg	0.95	0.94	0.94	53

**Fig. 4:**
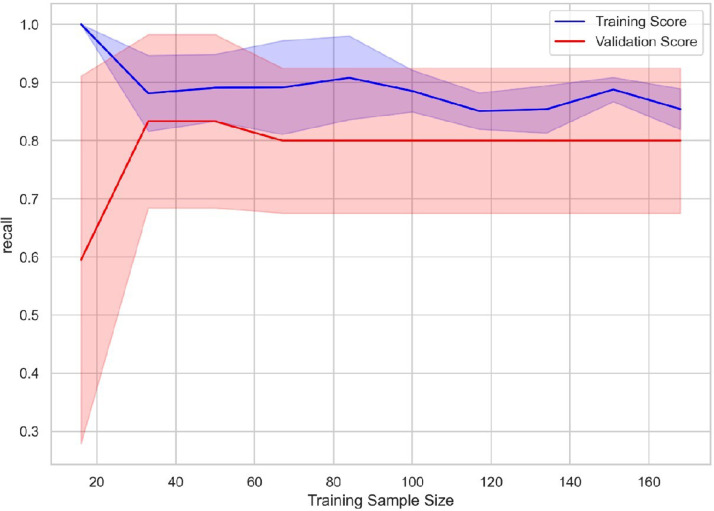
Learning curve.

The learning curve Fig. 4 illustrates the model’s stability across varying training set sizes, confirming its robustness and consistent performance with the available data.

**Fig. 5:**
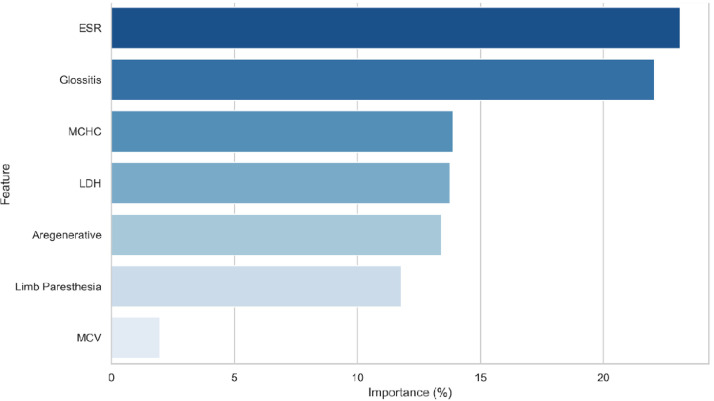
Relative feature importance based on the absolute values of logistic regression coefficient.

Finally, the relative importance of features contributing to the model’s predictions is visualized in Fig. 5. The most influential variables are ranked based on their contribution to the prediction of non-megaloblastic anemia.

Regarding feature importance, the analysis revealed that ESR, glossitis, and MCHC were the most influential variables in predicting non-megaloblastic anemia, with respective importance percentages of 23.11 %, 22.07 %, and 13.87 %. Other variables such as LDH (13.77 %), aregenerative status (13.42 %), and limb paresthesia (11.78 %) also contributed significantly to the model, while MCV had a comparatively lower importance (1.98 %). The variable importance was determined based on the absolute values of the logistic regression coefficients, reflecting their contribution to the model's predictions.

## 5 Discussion

### 5.1 Interpretation of results

To the best of our knowledge, this is the first study based on a ML approach to predict the non-megaloblastic origin of a macrocytic anemia without assessing vitamin B12 or folate levels and without performing a bone marrow aspiration. The performance of our logistic regression model in predicting non-megaloblastic anemia was satisfactory. The ROC curve demonstrated a high AUC, indicating strong discriminatory power. The optimal cutoff point was identified, balancing sensitivity and specificity effectively. The confusion matrix revealed that the model accurately identified the majority of non-megaloblastic anemia cases, with a low rate of false positives and false negatives. The classification report showed high precision, recall, and F1-score for both classes, suggesting that the model performs well across different evaluation metrics.

The learning curve analysis indicated that both training and validation scores converged and stabilized, suggesting that the model has been well-tuned and is not overfitting. This convergence implies that the model's performance is consistent across different subsets of the data, enhancing its generalizability.

Overall, the results demonstrate that our model is effective in predicting non-megaloblastic anemia, with robust performance metrics and a clear understanding of the contributing factors.

Machine learning, a subset of AI, offers an opportunity for integrating complex and diverse datasets, assisting therefore healthcare professionals in their decision-making endeavors, and offering a personalized healthcare, enhancing thereby therapeutic outcomes [5]. A spectrum of clinical, anatomopathological, radiological, and biological data, for both benign and malignant hematologic conditions such as blood transfusion, myelodysplastic syndrome, bone marrow failure syndromes, and acute myeloid leukemia have benefitted from AI [[5], [6], [7]].

Macrocytic anemia accounts for 6.3 % of men and 3.3 % of women among elderly [8]. Megaloblastic anemia, resulting from deficiency or impaired utilization of vitamin B12 and/or B9, may lead to irreversible neurologic complications especially in frail elderly if not diagnosed and treated timely. Indeed, vitamin B12 deficiency usually leads to neurological symptoms such as dysesthesia and paresthesia and in severe cases combined degeneration, thus limb paresthesia was found to contribute significantly to our model accounting for 11.78 %. Apart from the neurological symptoms, glossitis, also called Hunter glossitis, is attributable to vitamin B12 deficiency and contributed 22.07 % to our model. High levels of LDH are also reported in vitamin B12 and/or B9 deficiency resulting from impaired nuclear differentiation [9].

Reticulocytes count is an important parameter to evaluate in case of macrocytic anemia. In fact, reticulocytes are immature nonnucleated erythrocytes. Found in high levels, they should rule out hemolysis or blood loss. Megaloblastic anemia is usually non regenerative unless blood transfusion or vitamin supplementation [9]

Mean corpuscular volume over which megaloblastic anemia is highly likely was previously reported as 110, 115, 120 or 130 fl [[10], [11], [12], [13], [14]]. According to our results this parameter was of lower importance as it contributed 1.98 % to the model. This could be attributed to the fact that iron deficiency patients were not ruled out.

### 5.2 Study limitations

In this study, several limitations must be considered. The sample size is relatively small, which can affect the robustness and generalizability of the results. Additionally, the class imbalance, with a less represented target class, posed a significant challenge. Although appropriate techniques were employed to manage this imbalance, the absence of synthetic data generation could have limited the effectiveness of these methods. Patients with iron deficiency anemia were not excluded. This could have affected MCV levels, and consequently may explain the lower importance of this parameter in predicting non-megaloblastic anemia in our model.

In summary, despite promising results, these limitations suggest that future studies with larger samples, the integration of additional data, and the exploration of different class imbalance management techniques are necessary to improve the accuracy and clinical applicability of the model.

### 5.3 Implications for clinical practice

In resource-limited settings, accurately distinguishing between megaloblastic and non-megaloblastic macrocytic anemia is crucial. This differentiation enables healthcare providers to initiate appropriate treatments promptly. For instance, identifying megaloblastic anemia allows for the administration of vitamin B12 or folate supplements, which can be life-saving. Conversely, recognizing non-megaloblastic anemia, which may result from conditions like myelodysplastic syndromes, hemopathies or liver diseases, necessitates referral to specialized centers for advanced diagnostic evaluation and management. This timely referral is essential to prevent delays in addressing potentially serious underlying conditions.

Implementing a predictive model, such as the one developed in this study, can assist clinicians in making informed decisions regarding patient management. By accurately identifying patients with non-megaloblastic anemia, healthcare providers can prioritize referrals to specialized centers, ensuring that patients receive the necessary care without unnecessary delays.

### 5.4 Future research prospects

In the future, we plan to develop a web application that will enable our partners to apply the model in their clinical settings. Initially, the medical team will not rely on the model's results until it has been validated through rigorous clinical trials. To achieve this, we will conduct large-scale evaluations to assess the model's performance across diverse patient populations and clinical environments.

Subsequently, we aim to expand the sample size and retrain the model to determine whether this enhances its performance or alters the selection of significant variables. Increasing the sample size can improve the model's generalizability and robustness, as larger datasets often lead to more accurate and reliable predictions.

By systematically evaluating and refining the model, we strive to ensure its clinical applicability and effectiveness in real-world settings.

## 6 Conclusion

In conclusion, this study demonstrates the potential of using logistic regression models to predict non-megaloblastic macrocytic anemia in resource-limited settings. By developing a web application, we aim to facilitate the application of this model in regional hospitals, enabling early identification and appropriate management of patients. While the results are promising, further validation and refinement are necessary to enhance the model's accuracy and clinical applicability. Future research should focus on expanding the sample size and integrating additional data, thereby improving the model's performance and its potential impact on patient care.

## Funding

None.

## Informed consent

informed consent from patients and authorization for publication of the series has been obtained.

## CRediT authorship contribution statement

**Melek Kechida:** Conceptualization, Methodology, Writing – original draft. **Mohamed Kenani:** Data curation, Methodology, Writing – original draft. **Nader Slama:** Conceptualization, Methodology, Writing – original draft. **Manel Mili:** Formal analysis, Methodology, Software, Validation. **Aminetou Salem:** Writing – original draft. **Wassim Krit:** Data curation. **Ines Khochtali:** Validation. **Sarra Boukhris:** Writing – original draft. **Asma Ben Abdallah:** Methodology, Supervision. **Mohamed Hedi Bedoui:** Formal analysis, Supervision, Writing – review & editing.

## Declaration of competing interest

The authors declare that they have no conflict of interest.

## References

[1] Nagaro T., Hirokawa M. (2017). Diagnosis and treatment of macrocytic anemias in adults. J. Gen. Fam. Med..

[2] Haemoglobin concentrations for the diagnosis of anaemia and assessment of severity ». https://www.who.int/data/nutrition/nlis/info/anaemia.

[3] Takahashi N., Kameoka J., Takahashi N., Tamai Y., Murai K. (2016). Causes of macrocytic anemia among 628 patients: mean corpuscular volumes of 114 and 130 fL as critical markers for categorization. Int. J. Hematol..

[4] Radakovich N., Nagy M., Nazha A. (2020). Artificial Intelligence in hematology: current challenges and opportunities. Curr. Hematol. Malig. Rep..

[5] Maynard S., Farrington J., Alimam S., Evans H., Li K., Wong W.K., Stanworth S.J. (2024). Machine learning in transfusion medicine: a scoping review. Transfusion.

[6] D'Amico S., Dall'Olio D., Sala C., Dall'Olio L., Sauta E. (2023). Synthetic data generation by artificial intelligence to accelerate research and precision medicine in hematology. JCO Clin. Cancer Inform..

[7] Gutierrez-Rodrigues Fernanda, Munger Eric, Ma Xiaoyang, Groarke Emma M, Tang Youbao, Patel Bhavisha A (2023). Differential diagnosis of bone marrow failure syndromes guided by machine learning. Blood.

[8] Inelmen E.M., D’Alessio M., Gatto M.R. (1994). Descriptive analysis of the prevalence of anemia in a randomly selected sample of elderly people living at home: some results of an Italian multicentric study. Aging (Milano).

[9] Nagao Takayo, Hirokawa Makoto (2017). Diagnosis and treatment of macrocytic anemias in adults. J. Gen. Fam. Med..

[10] Babior B.M., Bunn H.F, Kasper D.L., Braunwald E., Hauser S., Longo D., Jameson J.L., Fauci A.S. (2005). Harrison’s Principles of Internal Medicine.

[11] Schnall S.F., Berliner N., Duffy T.P., Benz E.J, Hoffman R., Benz E.J., Shattil S.J., Furie B., Cohen H.J., Silberstein L.D. (2000). Hematology. Basic principles and Practice.

[12] Savage D.G., Ogundipe A., Allen R.H., Stabler S.P., Lindenbaum J. (2000). Etiology and diagnostic evaluation of macrocytosis. Am. J. Med. Sci..

[13] Aslinia F., Mazza J.J., Yale S.H. (2006). Megaloblastic anemia and other causes of macrocytosis. Clin. Med. Res..

[14] Takahashi Natsuko, Kameoka Junichi, Takahashi Naoto, Tamai Yoshiko, Murai Kazunori, Honma Riko (2016). Causes of macrocytic anemia among 628 patients: mean corpuscular volumes of 114 and 130 fL as critical markers for categorization. Int. J. Hematol..

